# Supplementary material for: A neural correlate of perceptual segmentation in macaque middle temporal cortical area
Source: Nat Commun. 2022 Aug 24;13:4967. doi: 10.1038/s41467-022-32555-y (PMC9402536; doi:10.1038/s41467-022-32555-y)
Supplement: Supplementary file 1 — Supplementary Information [file 41467_2022_32555_MOESM1_ESM.pdf]

SUPPLEMENTARY INFORMATION

Supplementary Figure 1

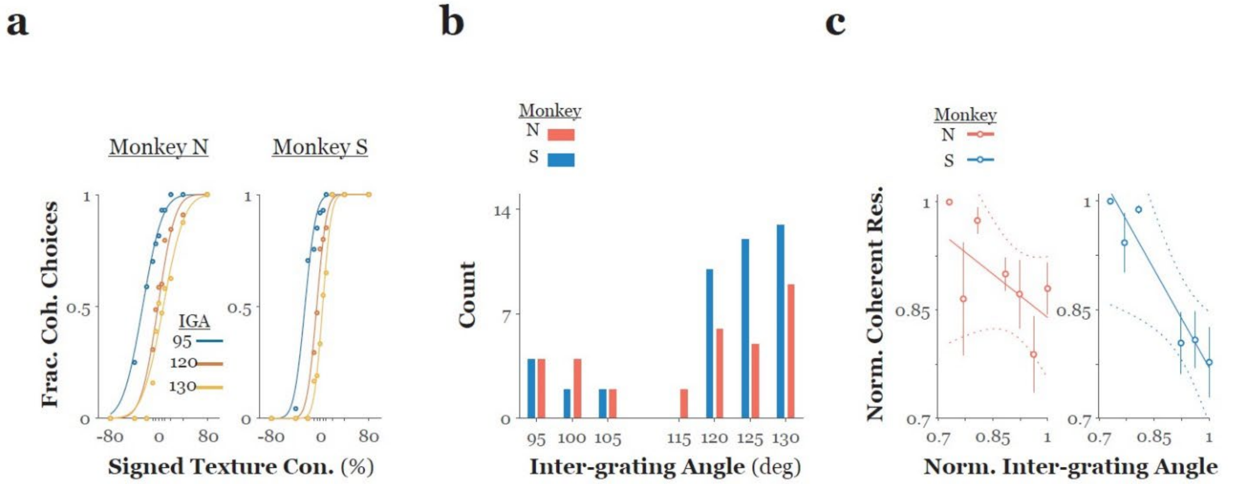

Supplementary Figure 1 – Effect of varying plaid inter-grating angle on plaid perception. (a) In each panel, perceptual judgements (fraction coherent choices - ordinate) are plotted versus plaid texture contrast (transparent cues assume negative values - abscissa). Psychometric functions from sessions with different inter-grating angles are plotted in separate colors; data from each monkey is plotted in a different panel (monkey N(S) – left (right)). Decreasing (increasing) inter-grating angle had a similar effect across animals, e.g. an increase (decrease) in the probability of a coherent judgment for a plaid pattern with a given texture contrast. **(b)** Number of sessions at each inter-grating angle for each monkey. **(c)** In each panel, the mean normalized coherent response (fraction coherent judgments/maximum) for zero-texture contrast plaids is plotted versus the normalized inter-grating angle. Varying plaid inter-grating angle had a qualitatively similar effect on perception of zero-texture contrast plaids across monkeys (Pearson's correlation; monkey N: r = -0.21, p = 0.05; monkey S: r = 0.35, p = 0.01) this effect was qualitatively similar to that seen for plaids with texture contrast (see Results and Fig. 2D). Error bars represent standard error of the mean. Sample sizes at each inter-grating angle are given in panel **(b)**. Frac., fraction; coh., coherent; con., contrast; deg, degrees; norm., normalized

Supplementary Figure 2

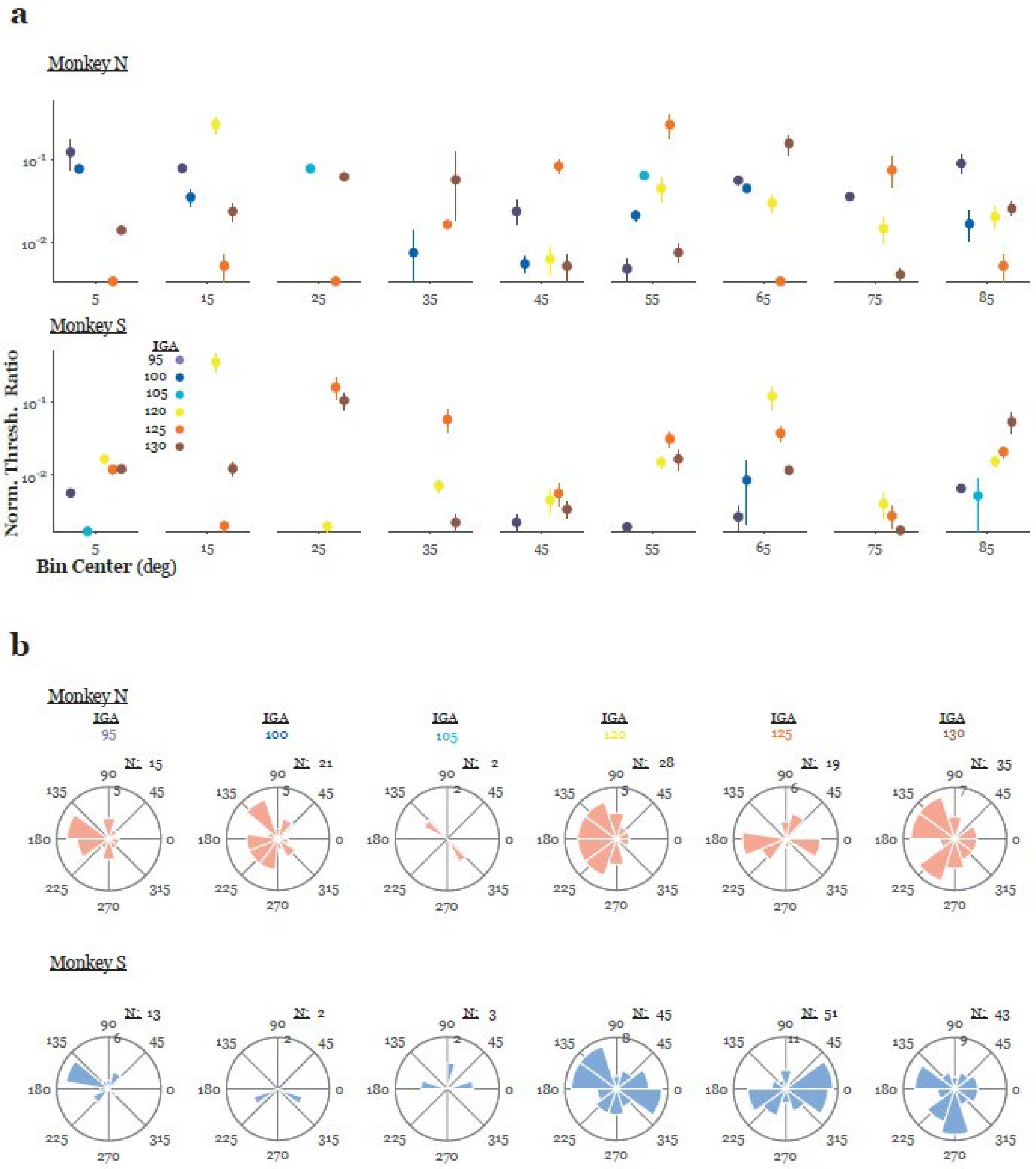

Supplementary Figure 2 – Effect of varying plaid inter-grating angle on neuronal sensitivity to plaid patterns (a) The normalized difference in neuronal sensitivity (normalized threshold ratio – ordinate) is plotted versus the difference between a unit's preferred direction and the nearest plaid pattern direction (defined as the "best" plaid pattern direction e.g., for a unit with a preferred direction of 45° the nearest plaid pattern direction would be 90°). Normalized preferred directions were binned (10° bins) and normalized threshold ratios (worst direction/best direction) were grouped by plaid inter-grating angle within each bin before averaging. Data from monkey N (S) are shown in the top (bottom) row. Data points representing different inter-grating angle conditions are shifted horizontally from the bin center in each plot for ease of visualization. Although there was a trend towards an effect of plaid inter-grating angle on the relationship between relative sensitivity and MT units' preferred direction in monkey S, this effect did not reach significance in either animal (ANOVA; monkey N: IGA – $F = 0.58$, $p = 0.71$, norm. pref. dir. – $F = 0.98$, $p = 0.46$, IGA x norm. pref. dir – $F = 0.77$, $p = 0.76$; monkey S: IGA – $F = 0.4$, $p = 0.85$, norm. pref. dir. – $F = 1.26$, $p = 0.27$, IGA X norm. pref. dir. – $F = 1.58$, $p = 0.07$). Error bars represent standard error of the mean. Sample sizes (single units) at each inter-grating angle are given above the rose histograms in panel **(b)** **(b)** Rose histograms of the preferred directions (measured with single sine gratings) for all units in the sample. Data in each column are from units tested with a particular plaid inter-grating angle (given above each column). Data from monkey N (S) are in the top (bottom) row. The number of units from each animal sampled with each inter-grating angle are given above each plot; for both monkeys the majority of data was collected with inter-grating angles between 120-130° (see Supp. Fig. 1b). For both monkeys, there was no consistent significant mean vector in the distribution of preferred directions for any of the inter-grating angle conditions (Rayleigh test; monkey N: IGA – 95, $z = 1.28$, $p = 0.28$, IGA - 100, $z = 1.84$, $p = 0.16$, IGA – 105, $z = 0.12$, $p = 0.9$, IGA – 120, $z = 2.38$, $p = 0.1$, IGA – 125, $z = 0.07$, $p = 0.92$, IGA – 130, $z = 1.41$, $p = 0.25$; monkey S: IGA – 95, $z = 3.14$, $p = 0.04$, IGA – 100, $z = 0.36$, $p = 0.74$, IGA – 105, $z = 0.37$, $p = 0.72$, IGA – 120, $z = 0.02$, $p = 0.97$, IGA – 125, $z = 1.74$, $p = 0.18$, IGA – 130, $z = 1.32$, $p = 0.27$) nor was there a significant difference in the distribution of preferred directions tested with each inter-grating angle (Watson-Williams; monkey N: $F = 0.37$, $p = 0.87$; monkey S: $F = 1.69$, $p = 0.14$). Norm., normalized; thresh., threshold; deg, degrees; IGA, inter-grating angle

Supplementary Figure 3

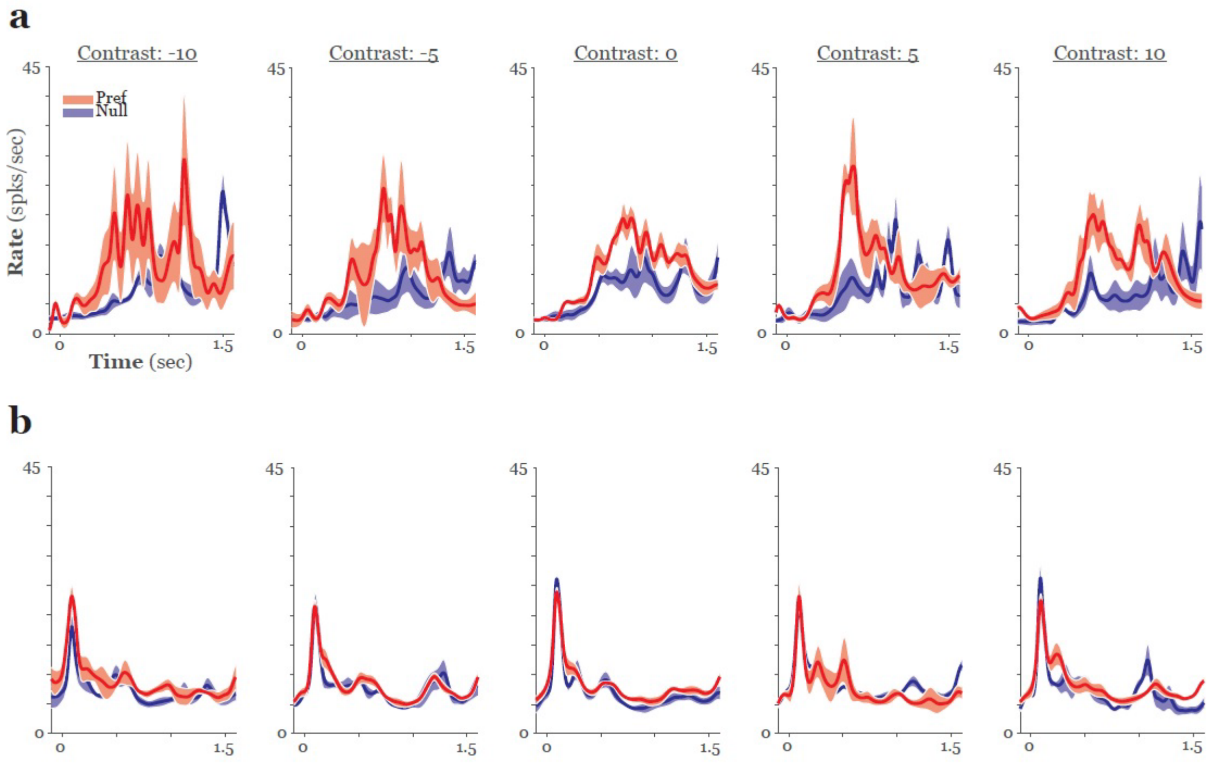

Supplementary Figure 3 – Example MT single-unit responses to a constant plaid stimulus sorted by perceptual report. (a) In each column, firing rate (ordinate) is plotted against time (abscissa) separately for trials that ended in a perceptual judgement that was congruent ("Pref" – red traces) or incongruent ("Null" – blue traces) with the example neuron's preferred plaid configuration. Plaid texture contrast goes from 10% transparent to 10% coherent from left to right (signed texture contrast denoted above each column), all plaids drifted in an upward pattern direction. This unit preferred coherent texture cues for upward drifting plaids, so the increased firing rate on trials that ended in a judgment congruent with this unit's preferred plaid configuration were on error (correct) trials for plaids with negative (positive) texture contrast. Data in each panel is taken from the same unit shown in the main text in Figures 3a, 4a, and 7b (top row). (b) Same conventions as in (a) but for the example MT unit shown in the main text in Figures 3b, 4b, and 7b (bottom row). Here, the stimulus was a downward drifting zero-texture contrast plaid. Spks, spikes; sec, second; pref., preferred

Supplementary Figure 4

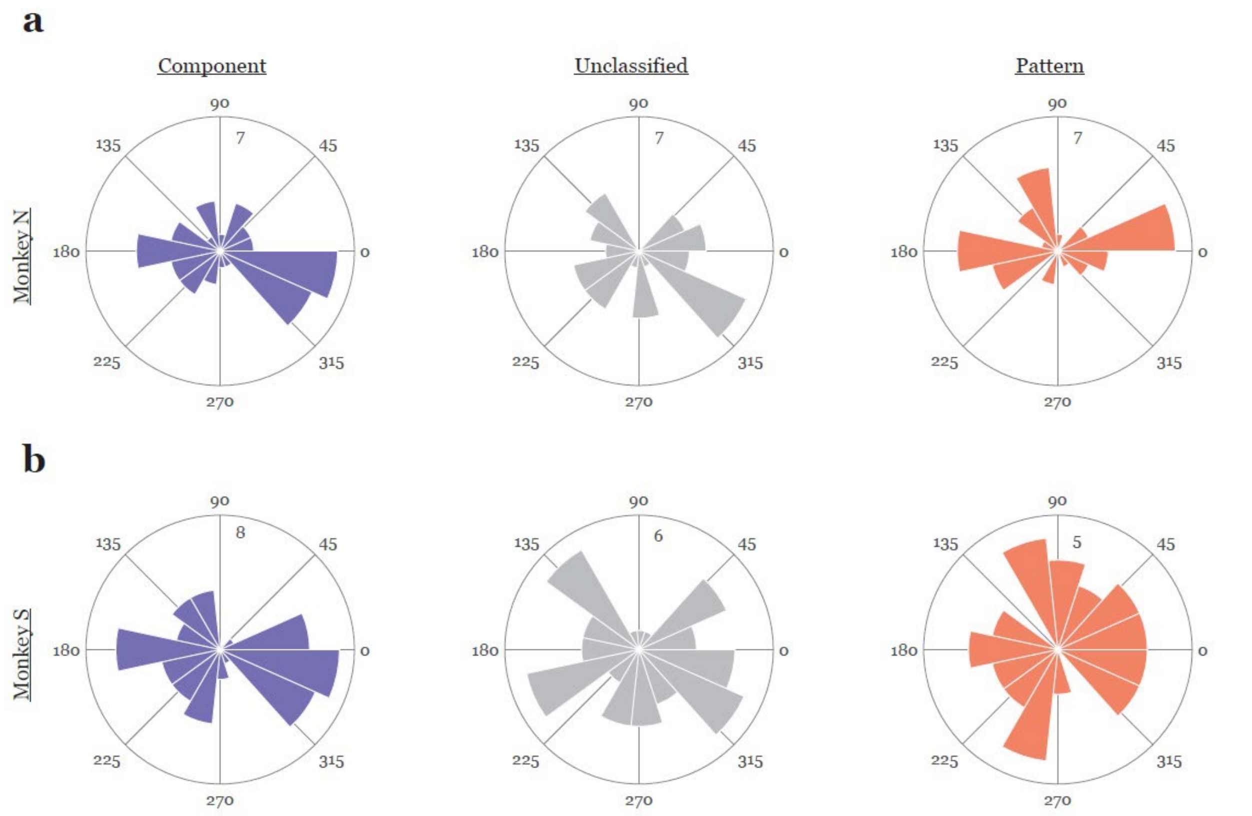

Supplementary Figure 4 – Distributions of MT single-unit preferred directions sorted by pattern-component classification. (a) Rose histograms of preferred directions (measured with single sine gratings) for all units recorded from monkey N that were classified as component (left), unclassified (middle), or pattern (right) cells. There was no significant mean vector in any of the distributions, nor was there a difference in the distributions across pattern-component classification (Rayleigh test; pattern cells – $z = 0.6$, $p = 0.55$, unclassified – $z = 1.55$, $p = 0.21$, component – $z = 0.16$, $p = 0.84$, Watson-Williams; $F = 1.87$, $p = 0.16$ **(b)** Same conventions as in **(a)** but for data from monkey S. Again, there was no significant difference in the distributions across pattern-component class (Rayleigh test, pattern cells – $z = 0.24$, $p = 0.79$, unclassified – $z= 0.96$, $p = 0.39$, component – $z = 0.89$, $p = 0.41$; Watson – Williams, $F = 2.34$, $p = 0.09$).
