# Supplementary material for: A neural correlate of perceptual segmentation in macaque middle temporal cortical area
Source: Nat Commun. 2022 Aug 24;13:4967. doi: 10.1038/s41467-022-32555-y (PMC9402536; doi:10.1038/s41467-022-32555-y)
Supplement: Supplementary file 3 — Description of Additional Supplementary Files [file 41467_2022_32555_MOESM3_ESM.pdf]

Description of Additional Supplementary Files

Supplementary Movie 1 – Example of bi-stable perception in square-wave plaid stimuli

The file contains a brief movie clip illustrating the two types of square-wave grating plaid stimuli used in the experiments. The clip begins with a plaid stimulus with a high contrast **coherent** texture cue (see Methods). The contrast of the cue is progressively lowered, until a plaid with no texture cue is presented. A series of drifting plaid stimuli with a variable contrast (from low to high) **transparent** texture cue is then presented. Here, the angle separating plaid components (inter-grating angle (IGA)) as well as the luminance of grating overlaps is set to maximize the variable perception of coherence and transparency. In the experiments, plaids were additive (e.g., the luminance of grating overlaps was twice that of each component grating) and the IGA was varied across sessions (see Methods for details of IGAs used for each monkey).
