# Supplementary material for: A neural correlate of perceptual segmentation in macaque middle temporal cortical area
Source: Nat Commun. 2022 Aug 24;13:4967. doi: 10.1038/s41467-022-32555-y (PMC9402536; doi:10.1038/s41467-022-32555-y)
Supplement: Supplementary file 5 — Reporting Summary [file 41467_2022_32555_MOESM5_ESM.pdf]

nature portfolio

Corresponding author(s): Andrew M. Clark

Last updated by author(s): Jul 22, 2022

Reporting Summary

Nature Portfolio wishes to improve the reproducibility of the work that we publish. This form provides structure for consistency and transparency in reporting. For further information on Nature Portfolio policies, see our Editorial Policies and the Editorial Policy Checklist.

Statistics

For all statistical analyses, confirm that the following items are present in the figure legend, table legend, main text, or Methods section.

n/a	Confirmed	
☐	☒	The exact sample size (n) for each experimental group/condition, given as a discrete number and unit of measurement
☐	☒	A statement on whether measurements were taken from distinct samples or whether the same sample was measured repeatedly
☐	☒	The statistical test(s) used AND whether they are one- or two-sided<br>*Only common tests should be described solely by name; describe more complex techniques in the Methods section.*
☐	☒	A description of all covariates tested
☐	☒	A description of any assumptions or corrections, such as tests of normality and adjustment for multiple comparisons
☐	☒	A full description of the statistical parameters including central tendency (e.g. means) or other basic estimates (e.g. regression coefficient) AND variation (e.g. standard deviation) or associated estimates of uncertainty (e.g. confidence intervals)
☐	☒	For null hypothesis testing, the test statistic (e.g. F, t, r) with confidence intervals, effect sizes, degrees of freedom and P value noted<br>*Give P values as exact values whenever suitable.*
☒	☐	For Bayesian analysis, information on the choice of priors and Markov chain Monte Carlo settings
☒	☐	For hierarchical and complex designs, identification of the appropriate level for tests and full reporting of outcomes
☒	☐	Estimates of effect sizes (e.g. Cohen's d, Pearson's r), indicating how they were calculated

Our web collection on statistics for biologists contains articles on many of the points above.

Software and code

Policy information about availability of computer code

Data collection	Multichannel Acquisition Processor (MAP) RASPUTIN, Version 1, Plexon Inc. (Dallas, Tx) - neural signal processing<br>Offline Sorter, Version 2.8.8, Plexon Inc. (Dallas, Tx) - single-unit spike sorting<br>Defunct Custom C Code (OhBehave/OhDisplay) - behavioral task control/behavioral event collection/visual stimulus display
Data analysis	No novel or non-standard methodology was used in the data analyses reported in this manuscript. All functions used for analysis were standard implementations in MATLAB 2017a, Mathworks Inc. (Natick, MA). All custom code used for data analysis is publicly available at: https://github.com/clarkam80/mtperceptualsegmentation-dataanalysis

For manuscripts utilizing custom algorithms or software that are central to the research but not yet described in published literature, software must be made available to editors and reviewers. We strongly encourage code deposition in a community repository (e.g. GitHub). See the Nature Portfolio guidelines for submitting code & software for further information.

Data

Policy information about availability of data

All manuscripts must include a data availability statement. This statement should provide the following information, where applicable:

- Accession codes, unique identifiers, or web links for publicly available datasets
- A description of any restrictions on data availability
- For clinical datasets or third party data, please ensure that the statement adheres to our policy

These data are currently being used to inform and validate an on-going computational modeling study; thus, they are not currently publicly available at this time but we are prepared to make them available upon reasonable request to the corresponding author.

Field-specific reporting

Please select the one below that is the best fit for your research. If you are not sure, read the appropriate sections before making your selection.

☒ Life sciences ☐ Behavioural & social sciences ☐ Ecological, evolutionary & environmental sciences

For a reference copy of the document with all sections, see nature.com/documents/nr-reporting-summary-flat.pdf

Life sciences study design

All studies must disclose on these points even when the disclosure is negative.

Sample size	A sample size of 2 animals is standard methodology in non-human primate systems neuroscience (cf. Fries & Maris (2021). arXiv).
Data exclusions	As described in the Methods, we excluded less than 20% of all behavioral sessions for performance that we could not reasonably conclude was under good stimulus control. We excluded 12% of all recorded single-units for either failure to maintain signal isolation throughout the duration of the experiment or insufficient sampling.
Replication	We repeated the behavioral task numerous times in each animal (monkey N – 36 sessions, monkey S – 50 sessions) to ensure the behavioral patterns were consistent within and across subjects. 4 (7) sessions in monkey N (S) failed to replicate (e.g., monkeys' trial-by-trial choices were not systematically affected by stimulus manipulations).
Randomization	There were no control or experimental groups as we did not employ any manipulations beyond the perceptual segmentation task, which was performed in both animals.
Blinding	No blinding was necessary, as there was no group allocation.

Reporting for specific materials, systems and methods

We require information from authors about some types of materials, experimental systems and methods used in many studies. Here, indicate whether each material, system or method listed is relevant to your study. If you are not sure if a list item applies to your research, read the appropriate section before selecting a response.

Materials & experimental systems

n/a	Involved in the study	
☒	☐	Antibodies
☒	☐	Eukaryotic cell lines
☒	☐	Palaeontology and archaeology
☐	☒	Animals and other organisms
☒	☐	Human research participants
☒	☐	Clinical data
☒	☐	Dual use research of concern

Methods

n/a	Involved in the study	
☒	☐	ChIP-seq
☒	☐	Flow cytometry
☒	☐	MRI-based neuroimaging

Animals and other organisms

Policy information about studies involving animals; ARRIVE guidelines recommended for reporting animal research

Laboratory animals	Adult rhesus macaques (macaca mulatta), one male (age 7 years) and one female (age 5 years), were used in this study.
Wild animals	No wild animals were used in this study.
Field-collected samples	No field-collected samples were used in this study.
Ethics oversight	The Institutional Animal Care and Use Committee at the University of Chicago approved all animal procedures. All animal research was designed and carried out in compliance with the Public Health Service's Guide for the Care and Use of Laboratory Animals.

Note that full information on the approval of the study protocol must also be provided in the manuscript.
